# To Cure Colds

**Published:** 1874-07

**Authors:** 


### TO CURE COLDS.

BY simply abstaining from drink and liquid food of any kind for as long a period as possible, the internal congestion—which is in fact the condition generally known as "a cold"—becomes reduced. The cause of congestion is the excess of blood contained in the overcharged membranes, and this is removed when the general bulk of the blood has been diminished by withholding the usual supply of fluid, by keeping the supply of drink for a day or two down to a point at which some degree of thirst is yet experienced, a complete cure may be effected.

